# The inhibition of the Aβ-ASC interaction site suppresses β-amyloid aggregation and cytotoxicity

**DOI:** 10.3389/fimmu.2026.1748748

**Published:** 2026-05-13

**Authors:** Lan Zhao, Xue Xia, Siqi Wang, Rui Liu, Zhenjiang Liu, Dong Sun, Xianghui Yu, Hui Wu

**Affiliations:** 1 National Engineering Laboratory for AIDS Vaccine, School of Life Sciences, Jilin University, Changchun, China; 2 Key Laboratory for Molecular Enzymology and Engineering, The Ministry of Education, School of Life Sciences, Jilin University, Changchun, China

**Keywords:** Alzheimer’s disease, ASC, Aβ aggregation, Aβ-ASC interaction, nanoparticle, β-amyloid

## Abstract

**Background and aims:**

The deposition of β-amyloid (Aβ) in the cerebral cortex and hippocampus is a key pathological hallmark of Alzheimer’s disease (AD). Previous studies have shown that ASC specks released by activated microglia can bind to Aβ and promote its aggregation, thereby accelerating AD progression. However, the specific mechanisms underlying this interaction remain poorly understood. This study aims to identify the interaction sites between ASC and Aβ, design a vaccine targeting these sites, and evaluate its immunogenicity and therapeutic efficacy.

**Methods:**

The interaction regions between ASC and Aβ were identified using pull-down and ELISA assays. Four types of nanoparticle carriers were purified using a prokaryotic expression system, and two peptides targeting the interaction sites were synthesized *in vitro*. These peptides were conjugated to the carriers via the SpyCatcher-SpyTag system. C57BL/6J mice were immunized with the constructed nanoparticles, and the immunogenicity of the vaccines was assessed by ELISA, while safety was evaluated by ELISpot. Serum antibodies were purified for further functional analysis.

**Results:**

Pull-down and ELISA results demonstrated that the C-terminal region of Aβ, particularly residues 29-42, and the pyrin domain (PYD) of ASC are key regions involved in binding. Eight nanoparticle vaccines carrying Aβ C-terminal epitopes were successfully prepared. Among them, the Ferritin-based Aβ vaccine induced a potent immune response targeting the Aβ-ASC interaction site without activating Aβ-specific T-cell responses. *In vitro* functional assays confirmed that the purified antibodies effectively disrupted Aβ-ASC binding, inhibited Aβ aggregation, and subsequently reduced its neurotoxicity.

**Discussion:**

Our findings indicate that the interaction site between ASC and Aβ is located within amino acids 29-42 of Aβ. Vaccines designed based on this site, Ferritin-Aβ29-35-3copy and Ferritin-Aβ36-42-3copy, effectively induced antibody production. The resulting antibodies suppressed Aβ aggregation and the neurotoxicity of Aβ oligomers. These data suggest that the Aβ-ASC interaction site may represent a potential target for AD therapy.

## 1 Introduction

Alzheimer’s disease (AD) is the leading cause of dementia worldwide, characterized by progressive deterioration in memory and cognitive functions, a gradual loss of the ability to perform daily activities, and the emergence of various neuropsychiatric symptoms and behavioral disturbances (1). Its primary pathological hallmarks include extracellular β-amyloid (Aβ) deposits, formed by the aggregation of Aβ peptides, and intraneuronal neurofibrillary tangles (NFTs), which consist of hyperphosphorylated Tau protein, along with neuroinflammation (2, 3). Extensive evidence implicates the pathological aggregation of Aβ as the primary catalyst for neuronal degeneration and cognitive decline (4, 5). Aβ peptides are generated proteolytically from the amyloid precursor protein (APP) via sequential cleavage by β- and γ-secretases (6, 7). Throughout the progression of AD, soluble Aβ monomers aggregate into neurotoxic oligomeric intermediates, which eventually form insoluble fibrillar aggregates (8, 9). These Aβ oligomers disrupt calcium homeostasis and compromise neuronal membrane integrity, ultimately leading to cell death (10, 11), while also inducing synaptic dysfunction and memory impairments (12, 13).

Apoptosis-associated speck-like protein containing a CARD (ASC), encoded by the PYCARD gene, plays a crucial role in innate immunity and inflammation. It is part of the death domain superfamily and contains both a pyrin domain (PYD) and a caspase recruitment domain (CARD) (14, 15). ASC is essential for the assembly of multiple inflammasomes, including NLRP3 (16, 17). Upon inflammasome activation, ASC polymerizes into ASC specks through PYD-mediated oligomerization and CARD-mediated cross-linking, forming platforms that activate caspase-1, drive the release of inflammatory cytokines, and ultimately induce pyroptosis (18). Moreover, there is growing evidence implicating ASC in the pathogenesis of various diseases, including cancer (19). Notably, accumulating evidence suggests that the microglial ASC protein serves as a pivotal regulator of Aβ aggregation. As the central adaptor of the NLRP3 inflammasome, ASC exhibits pathological activation in both AD patients and APP/PS1 transgenic models (20, 21), with increased serum ASC levels documented clinically (22). Activation of the NLRP3 inflammasome causes ASC to self-assemble into perinuclear specks, which are subsequently released into the extracellular space. These extracellular ASC specks act as heterologous nucleation sites, thereby accelerating Aβ aggregation (23). Notably, the knockout of ASC leads to reduced cerebral Aβ deposition and improved cognitive performance in mouse models (24, 25). In contrast, Aβ-ASC complexes exhibit dual pathological effects by intensifying neuroinflammation and triggering pyroptosis (26). These observations suggest that targeting the Aβ-ASC interaction could have therapeutic potential. However, the binding interface between ASC and Aβ remains to be characterized, and the efficacy of disrupting this interaction to prevent aggregation has not been experimentally validated.

To explore the molecular mechanism of the Aβ-ASC interaction and its therapeutic potential, we conducted a systematic investigation of the binding interface between ASC and Aβ, evaluating the functional consequences of disrupting this interaction. Utilizing *in vitro* binding assays, we identified Aβ29-42 as the crucial epitope mediating ASC binding. To target this structural domain, we engineered nanoparticle vaccines for mouse immunization, which successfully induced an antibody response against the C-terminal region of Aβ. We further isolated epitope-specific blocking antibodies from the immunized sera. Functional characterization demonstrated that these antibodies effectively inhibited the formation of the Aβ-ASC complex and impeded Aβ aggregation. Moreover, these antibodies protected neuronal cells by inhibiting the toxic effects of Aβ oligomers. Collectively, our study reveals the molecular basis of the Aβ-ASC interaction and suggests that targeting this interaction interface could represent a promising therapeutic strategy, providing novel insights for the treatment of Alzheimer’s disease.

## 2 Materials and methods

### 2.1 Animals

Female C57BL/6J wild-type mice, aged 6 to 8 weeks, were obtained from Liaoning Changsheng Biotechnology (Liaoning, China). All animals were housed in individually ventilated cage (IVC) systems at the Animal Experimental Platform, Core Facilities for Life Science, Jilin University. The experimental protocols received approval from the Institutional Animal Care and Use Committee of Jilin University (Approval No. [LACCAES2023008]). Housing conditions were carefully controlled, maintaining a temperature of 22 ± 1 °C, humidity at 50 ± 5%, and a specific pathogen-free (SPF) environment with a 12-hour light/dark cycle. All animal procedures in this study adhered to the U.S. National Research Council’s Guide for the Care and Use of Laboratory Animals and China’s Guidelines for Animal Welfare and Ethical Review.

### 2.2 Construction and purification of proteins

The full-length ASC gene was cloned into the pET-20b (+) expression vector to generate a recombinant plasmid for protein expression. This plasmid was transformed into competent E. coli cells, and protein expression was induced at 18 °C. After induction, bacterial cells were harvested and lysed by sonication, followed by centrifugation to collect the insoluble fraction. To extract the recombinant protein from inclusion bodies, the pellet was resuspended in 2 M guanidine hydrochloride and gently stirred at 4 °C for 2 hours to facilitate partial denaturation and solubilization. The sample was then centrifuged, and the supernatant was dialyzed against Tris-NaCl buffer for 16 hours to remove the denaturant and promote protein refolding. The refolded protein was further purified using a HisTrap™ HP affinity column (GE Healthcare, Chicago, USA) with a linear imidazole gradient from 25 to 500 mM. Fractions containing the target protein were collected and dialyzed overnight against Tris-NaCl buffer at 4 °C to obtain purified full-length ASC protein.

The gene encoding the ASC truncated variant was inserted into the pET-28a (+) vector to construct the expression plasmid. This truncated protein was expressed in soluble form. After sonication and centrifugation of the bacterial culture, the supernatant was directly applied to a HisTrap™ HP affinity column (GE Healthcare, Chicago, USA). The target protein was eluted using a linear gradient of 50-500 mM imidazole, and relevant fractions were collected.

The genes encoding the four nanoparticle carriers, namely AP205-SpyCatcher, Lus-SpyCatcher, HBc-SpyCatcher, and Ferritin-SpyCatcher, were individually cloned into the pET-28a (+) vector. The three His-tagged proteins, AP205-SpyCatcher, Lus-SpyCatcher, and HBc-SpyCatcher, were purified from the clarified supernatants using a HisTrap™ HP column (GE Healthcare, Chicago, USA) under identical elution conditions. In contrast, Ferritin-SpyCatcher was isolated from the supernatant by precipitation with saturated ammonium sulfate. The resulting pellet was dissolved and further purified using a Q ion-exchange column (GE Healthcare, Chicago, USA), eluted with a linear gradient of 50-500 mM NaCl to obtain the purified target protein.

### 2.3 Pull-down assay

To validate the interaction interface between ASC and Aβ, purified recombinant ASC protein was incubated with synthetic Aβ peptides (GenScript, Nanjing, China) in PBS buffer at 4 °C overnight, with constant rotation. The following day, 30 μL of Protein G agarose beads (Sigma-Aldrich, St. Louis, USA) were equilibrated by washing three times with 1× PBS. The beads were then incubated with 3.0 μg of rabbit anti-ASC polyclonal antibody (Proteintech, Cat# 10500-1-AP, RRID: AB_2174862) in 200 μL PBS at 4 °C for 1 hour with constant rotation to facilitate antibody coupling. After three additional washes with 1×PBS to remove unbound antibodies, the antigen mixture containing ASC and Aβ was added to the antibody-conjugated beads and incubated at 4 °C for 2 hours. Subsequently, the beads were washed six times with a wash buffer (20 mM Tris-HCl, 100 mM NaCl, 0.1 mM EDTA, 0.05% Tween-20, pH 7.5) to eliminate nonspecifically bound proteins. The immunoprecipitated complexes were then eluted by resuspending the beads in 1× SDS loading buffer and boiling at 100 °C for 15 minutes. The eluates were subjected to Western blot analysis to detect co-precipitated Aβ.

For antibody blocking experiments, Aβ peptides were pre-incubated with purified serum antibodies at 4 °C for 4 hours before the addition of ASC protein. The mixture was then incubated overnight at 4 °C. The subsequent steps, including immunoprecipitation with ASC-specific Protein G beads and Western blot detection, were performed as described above.

### 2.4 Coomassie brilliant blue staining

Following SDS-PAGE, the polyacrylamide gel was carefully excised from the electrophoresis apparatus and subjected to protein staining. The gel was immersed in Coomassie Brilliant Blue staining solution and incubated at room temperature for 30 minutes to ensure uniform dye penetration and protein binding. After staining, the solution was discarded, and the gel was briefly rinsed with deionized water to remove any superficial dye. Destaining was performed by incubating the gel in a destaining solution, with periodic replacement of the solution, until the background was clear and the protein bands were distinctly visible. The stained gel was then digitally documented for further analysis.

### 2.5 Western blot

Subsequent to electrophoresis, proteins were transferred onto 0.2 μm nitrocellulose membranes (Cytiva, Marlborough, USA). The membranes were then blocked with 5% (w/v) skim milk in PBS at ambient temperature for 1 hour. Following the blocking step, the membranes were incubated with primary antibodies (detailed below) diluted in PBS containing 1% (w/v) skim milk, either at 4 °C overnight or at room temperature for 2 hours. The membranes underwent three washes with PBST (PBS containing 0.1% Tween-20) and a single wash with PBS, each lasting 5 minutes. Horseradish peroxidase (HRP)-conjugated secondary antibodies were prepared in PBS with 1% (w/v) skim milk and incubated with the membranes at room temperature for 1 hour. The secondary antibodies used included goat anti-rabbit IgG (1:10,000; Proteintech, Cat# SA00001-2, RRID: AB_2722564), goat anti-mouse IgG (1:10,000; Proteintech, Cat# SA00001-1, RRID: AB_2722565), and goat anti-human IgG (1:10,000; Proteintech, Cat# SA000017-1, RRID: AB_2890979). Following the repetition of the washing steps, enhanced chemiluminescence (ECL) substrate (MeilunBio, Liaoning, China) was applied, and protein bands were visualized using a Tanon-5200 imaging system (Tanon, Shanghai, China). The primary antibodies used in this study were as follows: Rabbit anti-ASC (1:1,000; Proteintech, Cat# 10500-1-AP, RRID: AB_2174862), Mouse anti-Aβ (1:1,000; BioLegend, Cat# 803001, RRID: AB_2564653), Rabbit anti-D3E10 (1:1,000; Cell Signaling Technology, Cat# 12843, RRID: AB_2798041), 3D6 antibody (GenScript, Nanjing, China).

### 2.6 Preparation of nanoparticle vaccines

To conjugate the synthesized epitope peptides SpyTag-Aβ29-35-3copy and SpyTag-Aβ36-42-3copy to the purified nanoparticle carriers for vaccine preparation, the peptides were diluted to 1 mg/mL using Tris-KCl buffer. The four types of purified nanoparticle carriers were quantified using the BCA method. The carriers and epitope peptides were then mixed at a molar ratio of 1:4 and incubated at 4 °C overnight. After the conjugation reaction, the products were validated using Coomassie Brilliant Blue staining and Western Blot to assess the conjugation and its efficiency.

### 2.7 Immunization and bleeding

Female C57BL/6J mice, aged 6 to 8 weeks and weighing 18-20 g, were randomly assigned to 14 groups, with 8 mice in each group, to evaluate the immunogenicity of the nanoparticle vaccines. Immunizations were administered via the intramuscular (i.m.) route every two weeks for a total of four doses. The immunization components included a protein antigen at 0.625 μmol/mouse, a CpG adjuvant at 10 μg/mouse, and a ZMF59 adjuvant at 50 μL/mouse.

Blood samples were collected via retro-orbital bleeding 24 hours prior to each immunization. The blood was allowed to clot at 37 °C for 1-2 hours, followed by centrifugation at 4,000 rpm for 15 minutes to separate the serum. All serum samples were aliquoted and stored at -20 °C until further analysis.

### 2.8 ELISA

To measure the serum antibody titers, ELISA plates were coated with 100 μL Aβ solution (1 μg/mL) per well. Immunized mouse serum was serially diluted four-fold across four gradients (1:200 to 1:12,800) for antibody incubation. HRP-conjugated goat anti-mouse IgG secondary antibody (1:10,000; Proteintech, Cat# SA00001-1, RRID: AB_2722565) diluted in PBS with 1% BSA (w/v) was added (100 μL/well). TMB substrate (TIANGEN, Beijing, China) was added for 25 min at room temperature (protected from light), and reactions were terminated with 2 M H_2_SO_4_. Absorbance was immediately measured at 450 nm using a microplate reader (Bio-Rad; Hercules, USA).

To investigate the binding between ASC and Aβ, ELISA plates were coated overnight with 100 μL/well of different Aβ solutions (5 μg/mL). Purified ASC protein was serially diluted four-fold across 12 gradients (starting from 20 μg/mL). ASC primary antibody (1:1,000; Proteintech, Cat# 10500-1-AP, RRID: AB_2174862) diluted in 1% BSA solution was added (100 μL/well), followed by HRP-conjugated secondary antibody incubation and TMB detection.

### 2.9 ELISpot cytokine assay

An interferon-γ (IFN-γ) enzyme-linked immunospot (ELISpot) assay was conducted to evaluate T cell responses to Aβ. Four weeks after the final immunization, mice were euthanized by CO_2_ inhalation, with death confirmed by cervical dislocation. Splenocytes were isolated through mechanical dissociation of the spleens and seeded into ELISpot plates (BD, Cat# 551881, RRID: AB_2868948) at a density of 1×10^6 cells per well. The stimulation conditions included Aβ peptides and Ferritin-SpyCatcher protein, both at a final concentration of 100 ng/mL. RPMI-1640 medium served as the negative control, while concanavalin A (ConA; Invitrogen, Carlsbad, USA) was used as the positive control. All procedures were performed in accordance with the manufacturer’s instructions (BD Biosciences).

### 2.10 Purification of immune serum antibodies

A volume of 200 μL of immune serum was thoroughly mixed with 300 μL of 0.9% NaCl. The mixture was then placed on ice, and 500 μL of saturated ammonium sulfate solution was added dropwise with gentle mixing to ensure uniform protein precipitation under cold conditions. After the complete addition, the sample was incubated at 4 °C for 1 hour to allow adequate precipitation, followed by centrifugation at 4 °C to collect the precipitate. The precipitate was carefully resuspended in 500 μL of 0.9% NaCl and returned to the ice. Subsequently, 250 μL of saturated ammonium sulfate solution was slowly added dropwise with mixing. The sample was incubated again at 4 °C for 1 hour and centrifuged. This precipitation step was repeated one to three times, as needed, to enhance the purity of the target components. Finally, the precipitate was dissolved in 200 μL of 0.9% NaCl for subsequent experimental use.

### 2.11 Preparation of Aβ monomers, Aβ oligomers, and Aβ fibrils

Aβ monomers were prepared by following established protocols (27). Briefly, 1 mg of Aβ peptide (GL Biochem, Shanghai, China) was dissolved in 2 mL of ice-cold hexafluoroisopropanol (HFIP) and incubated at 25 °C for 2 hours. After evaporating the HFIP, the peptide film was redissolved in 2 mL of HFIP, and this step was repeated three times. The resulting film was then dissolved in 0.5 mL of 2 mM NaOH, followed by ultrasonication and centrifugation to obtain Aβ monomers.

Aβ oligomers were prepared by dissolving 1 mg of synthetic Aβ peptide in 110 μL of HFIP and incubating it at room temperature for 1 hour in the dark. The solution was then aliquoted into 55 μL portions. After evaporation of the HFIP, the peptide film was dissolved in 10 μL of DMSO, mixed with 500 μL of DMEM, and incubated at 4 °C for 24 hours to obtain Aβ oligomers.

To prepare Aβ fibrils, 1 mg of synthetic Aβ peptide was dissolved in 100 mM NaOH and subjected to water bath sonication for 30 seconds to create an Aβ stock solution. The solution was then supplemented with 10 mM HEPES, 100 mM NaCl, and 0.02% NaN3, and incubated at 37 °C for 21 days to form Aβ fibrils.

### 2.12 Aggregation inhibition assay with purified serum antibodies

To assess the inhibitory effect of purified serum antibodies on Aβ aggregation, synthetic Aβ peptides were monomerized, purified ASC protein was incubated with shaking at 37 °C for 1 hour. Subsequently, the mixture containing ASC specks, Aβ monomers, and purified serum antibodies was incubated with shaking at 37 °C for 24 hours. Following incubation, samples were prepared for Western blot analysis.

### 2.13 CCK8

To assess cell viability, SH-SY5Y human neuroblastoma cells were seeded in a 96-well plate at a density of 2,000 cells per well and cultured at 37 °C for up to 24 hours to facilitate cell attachment. Following this, the culture medium was supplemented with 40 μg of Aβ oligomers and the corresponding purified serum antibodies, and the cells were incubated for an additional 48 hours at 37 °C. After treatment, 10 μL of CCK-8 solution (Beyotime Biotechnology, Shanghai, China) was added to each well, and the plate was incubated at 37 °C for 0.5 to 4 hours. The optimal incubation duration was determined by monitoring color development. Finally, the absorbance of each well was measured at a wavelength of 450 nm using a microplate reader to assess cellular metabolic activity and viability.

SH-SY5Y human neuroblastoma cells were seeded in 96-well plates at a density of 2,000 cells per well and cultured for 24 hours. The cells were then treated with 20 μg of Aβ monomers, ASC specks, and the corresponding purified serum antibodies for 24 hours at 37 °C. Following treatment, 10 μL of CCK-8 solution was added to each well and incubated for 0.5 to 4 hours at 37 °C. Absorbance was measured at 450 nm using a microplate reader to assess cell viability.

### 2.14 Immunofluorescence

Frozen brain sections (40 μm) from 12-month-old APP/PS1 mice were thawed at room temperature for 20 minutes, washed with TBS, and blocked for 2 hours in 3% BSA containing 0.3% Triton X-100. The sections were then incubated overnight at 4 °C with purified primary antibodies diluted in 3% BSA. Following TBS washes, the sections were incubated with Alexa Fluor^®^ 555-conjugated goat anti-mouse IgG (1:1000; Jackson ImmunoResearch, Cat# 115-565-146, RRID: AB_3095443) for 2 hours at room temperature in the dark. After mounting with an anti-fading medium (Beyotime Biotechnology, Shanghai, China), images were acquired using a confocal microscope (Zeiss) with a 20× objective.

### 2.15 Statistical analysis

Statistical analysis was conducted using GraphPad Prism version 10.4. Data are presented as the mean ± standard error of the mean (SEM). The interaction assessed by ELISA was analyzed using two-way analysis of variance (ANOVA), while an unpaired, two-tailed Student’s t-test was used for comparisons between two groups. A p-value < 0.05 was considered statistically significant. Western blot bands were quantified using ImageJ, and the graphical abstract was created with BioRender.com.

## 3 Results

### 3.1 The interaction interface between ASC and Aβ is localized within the C-terminal region of Aβ

**Figure 1:**
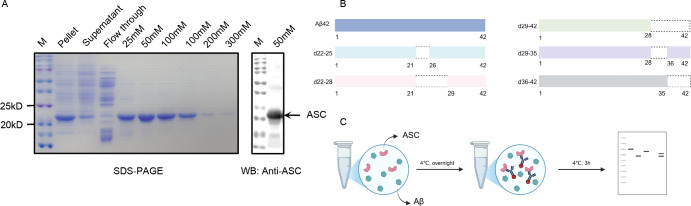
Purification of recombinant ASC protein and design of truncated Aβ peptides. **(A)** Characterization of the purified ASC protein. The eluted fractions were validated through SDS-PAGE (left) and immunoblotting with an anti-ASC antibody (right). **(B)** Schematic diagram of truncated Aβ peptide variants. Specifically, Aβ42 represents the full-length form; d22-25 lacks amino acid residues 22 to 25; d22-28 lacks residues 22 to 28; d29-42 lacks residues 29 to 42; d29-35 lacks residues 29 to 35; d36-42 lacks residues 36 to 42. **(C)** Schematic workflow of the pull-down assay. Complexes of recombinant ASC with various Aβ peptides were immunoprecipitated with an anti-ASC antibody-conjugated protein G beads and analyzed by Western blot.

**Figure 2:**
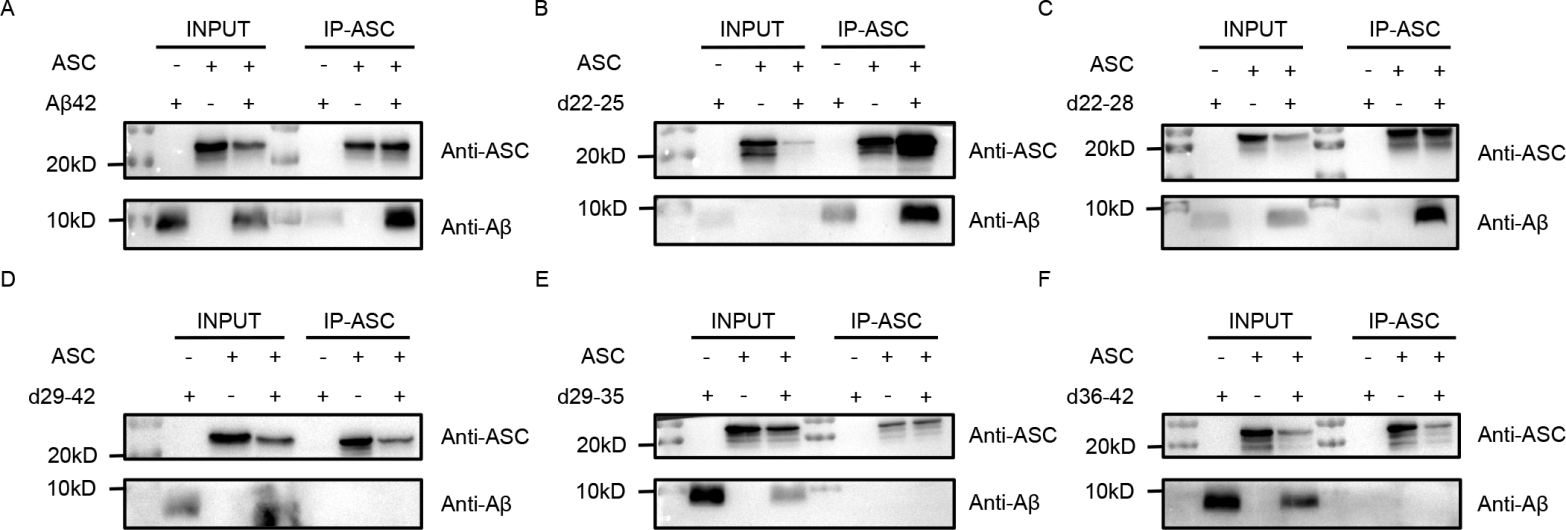
Identification of the ASC binding site on Aβ. **(A)** Validation of the interaction between ASC and full-length Aβ by pull-down assay. **(B-F)** The ASC-binding domain on Aβ was mapped through pull-down assays with truncated fragments d22-25 **(B)**, d22-28 **(C)**, d29-42 **(D)**, d29-35 **(E)**, and d36-42 **(F)**.

To investigate the interaction domains between ASC and Aβ, we first purified the ASC protein, which was expressed with a His-tag in a prokaryotic system, using Ni-affinity chromatography. The ASC protein was effectively eluted at a concentration of 50 mM imidazole. The identity and purity of the recombinant ASC protein were confirmed by Coomassie brilliant blue staining and Western blot analysis using an anti-ASC antibody (**Figure 1A**). We then systematically designed and synthesized a series of Aβ peptides. Specifically, the d22-25 peptide was characterized by the absence of residues 22-25, the d22-28 variant lacked residues 22-28, the d29-42 peptide was devoid of residues 29-42, the d29-35 variant had residues 29-35 removed, and the d36-42 peptide featured the deletion of residues 36-42 (**Figure 1B**). Subsequently, pull-down assays were performed to validate the interaction sites between ASC and Aβ. As detailed in **Figure 1C**, we utilized ASC-specific gel beads to capture the ASC protein, followed by Western blot analysis to assess the binding between ASC and the various Aβ peptides. Our pull-down assays successfully detected a robust interaction between ASC and the full-length Aβ peptide (**Figure 2A**). Furthermore, the deletion of Aβ residues 22-25 or 22-28 did not impair the binding to ASC (**Figures 2B, C**). In contrast, the deletion of residues 29-42 completely abolished the Aβ-ASC interaction (**Figure 2D**), suggesting that the critical interface is the C-terminal 29-42 region of Aβ. To precisely map the exact interaction site, we further employed the truncated peptides d29-35 and d36-42. Notably, the deletion of either residues 29-35 or 36-42 significantly impaired the Aβ-ASC interaction, confirming that both segments are involved in the binding (**Figures 2E, F**). In summary, these findings demonstrate that the C-terminal region of Aβ, spanning residues 29 to 42, is essential for mediating its interaction with ASC, with both the 29-35 and 36-42 fragments contributing critically to this interface.

### 3.2 The interaction interface between ASC and Aβ is located within the PYD domains of ASC

**Figure 3:**
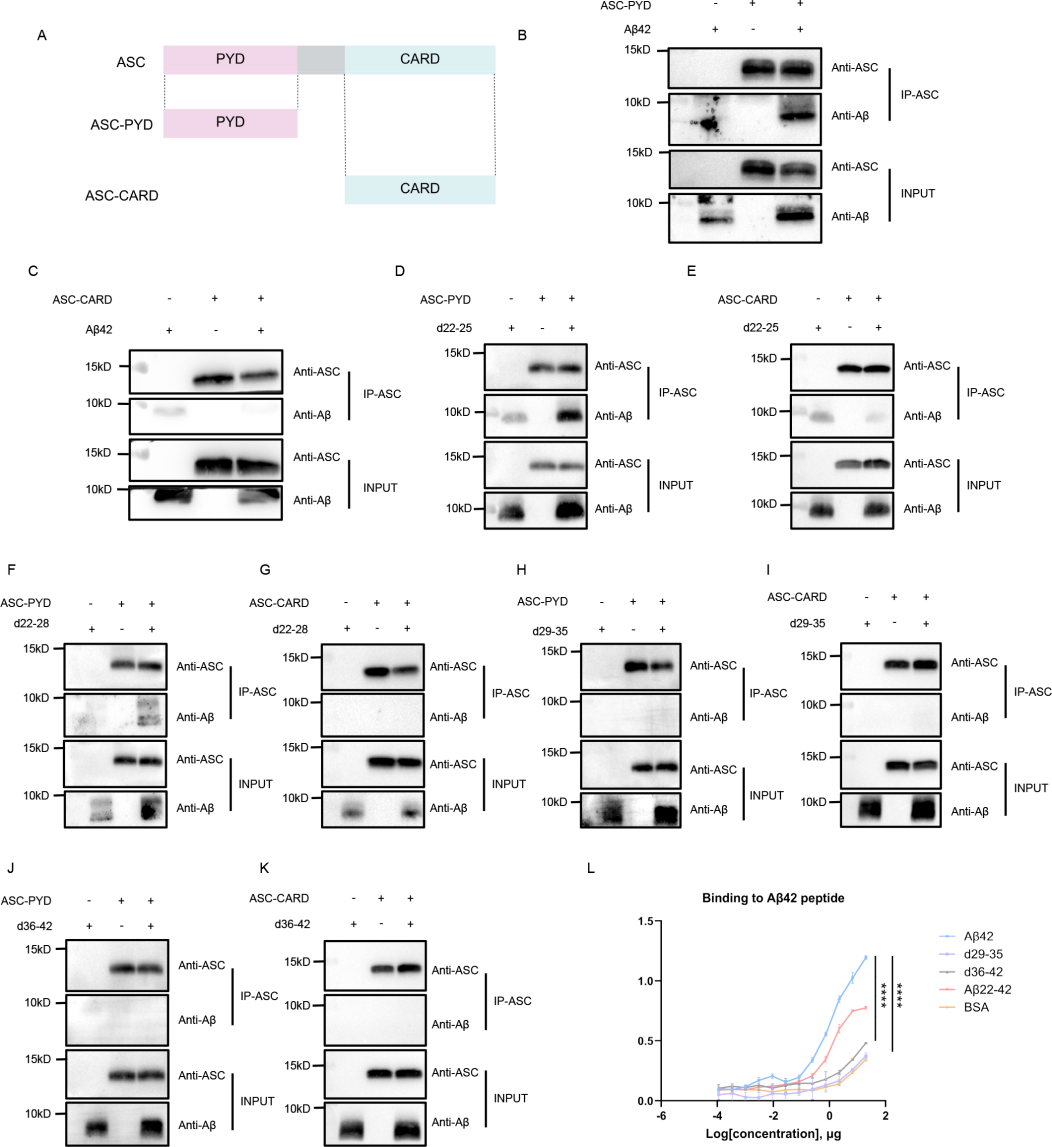
Determination of the Aβ binding site on ASC. **(A)** Schematic illustrations of truncated ASC proteins. **(B–C)** Detection of the interactions between full-length Aβ and the recombinant proteins ASC-PYD **(B)** and ASC-CARD by pull-down assays **(C)**. **(D–K)** Analysis of interactions between the ASC truncated domains (PYD and CARD) and various Aβ fragments, including ASC-PYD with d22-25 **(D)**, ASC-CARD with d22-25 **(E)**, ASC-PYD with d22-28 **(F)**, ASC-CARD with d22-28 **(G)**, ASC-PYD with d29-35 **(H)**, ASC-CARD with d29-35 **(I)**, ASC-PYD with d36-42 **(J)**, and ASC-CARD with d36-42 **(K)**. **(L)** Evaluation of the interaction between ASC and truncated Aβ peptides using ELISA. The data are presented as mean ± SEM (n = 2), and the data was analyzed using two-way ANOVA analysis, ****p < 0.0001.

The ASC protein features an N-terminal PYD domain and a C-terminal CARD domain, each with unique roles (**Figure 3A**). The PYD domain connects with upstream sensors and self-oligomerizes to create ASC filaments, while the CARD domain promotes caspase-1 oligomerization and activation (28). Thus, we also determined the ASC domain responsible for the interaction with Aβ. Through pull-down assays, we identified a direct interaction between the PYD domain and Aβ, while the CARD domain showed no binding affinity for Aβ (**Figures 3B, C**). Further pull-down experiments employing truncated variants of both ASC and Aβ were conducted to validate the interaction sites. The findings revealed that the Aβ peptide fragments d22-25 and d22-28 specific maintain the ability to interact with the PYD domain (**Figures 3D, F**), but not with the CARD domain (**Figures 3E, G**). Consistent with previous observations, the deletion of Aβ29-35 and Aβ36-42 fragment fully ruined the association of Aβ peptide with the PYD or CARD domain (**Figures 3H-K**). Additionally, ELISA results corroborated that Aβ22-42 binds to ASC with an affinity comparable to that of full-length Aβ, whereas truncated Aβ peptides lacking residues 29-35 or 36-42 exhibited reduced binding to ASC (**Figure 3L**). Collectively, these findings demonstrate that the PYD domain of ASC mediates the interaction with Aβ29-42 epitope.

### 3.3 Development of nanoparticle vaccines targeting the Aβ-ASC interaction interface

**Figure 4:**
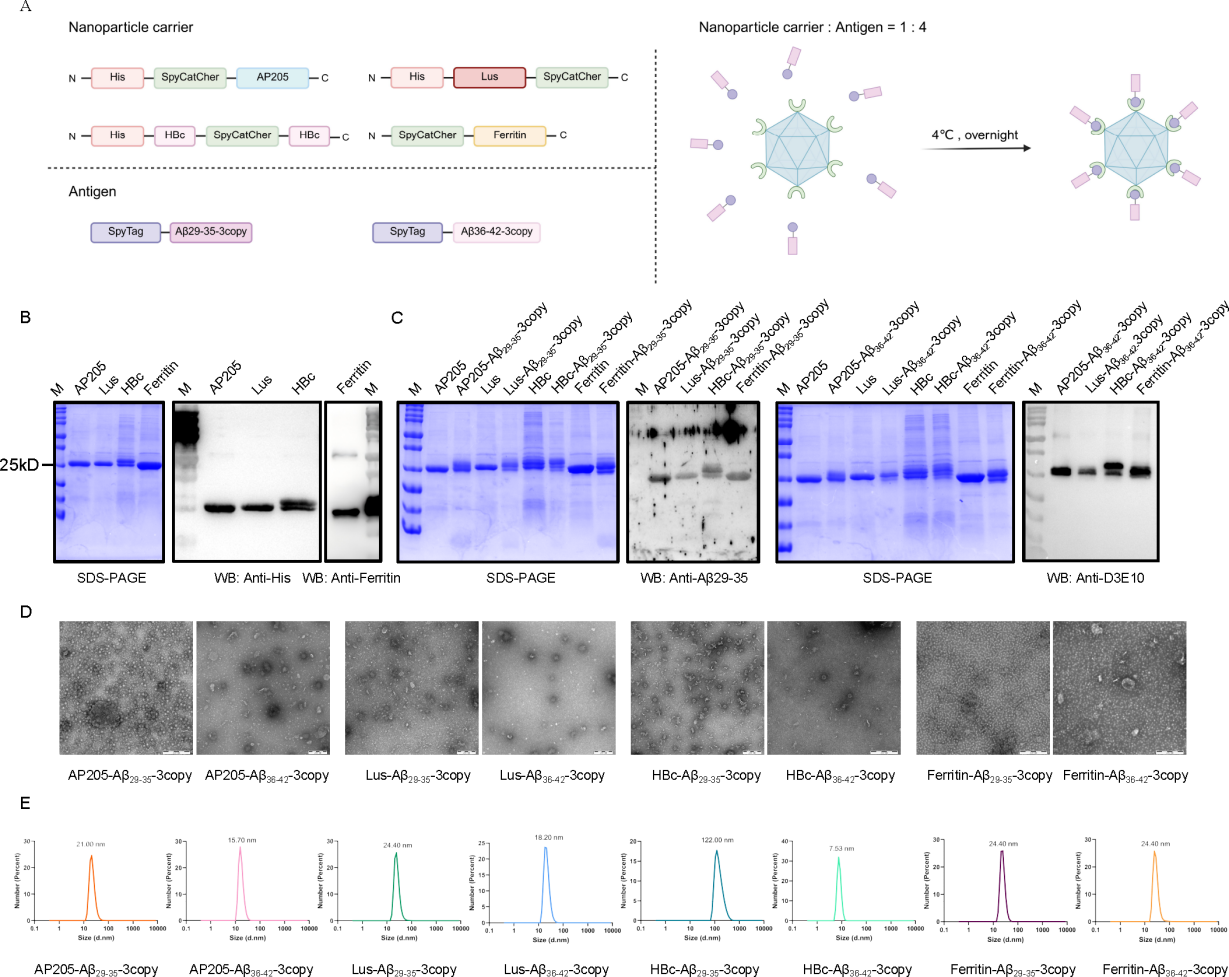
Preparation and characterization of nanoparticle vaccines targeting Aβ-ASC binding interface. **(A)** Schematic of four nanoparticle carriers and two antigen peptides (left). A schematic illustrating the *in vitro* conjugation via the SpyCatcher-SpyTag system (right). **(B)** Validation of nanoparticle carrier proteins. The purity of these proteins was verified by SDS-PAGE (left) and Western blot (right). **(C)** Characterization of recombinant nanoparticle vaccines after peptide conjugation. The conjugated vaccine was confirmed by Coomassie Blue staining (left) and Western blot (right). **(D)** A representative TEM image of distinct nanoparticles targeting Aβ29-35 or Aβ36-42 (scale bars = 200 nm). **(E)** Particle size analysis of the eight nanoparticles.

To effectively disrupt the Aβ-ASC interaction, we developed nanoparticle-based vaccines targeting the key binding sites on ASC and Aβ. These vaccines incorporate the previously identified Aβ29-35 and Aβ36-42 segments as core immunogens to elicit a specific antibody response. Nanoparticles serve as a highly efficient antigen delivery platform, offering significant advantages in enhancing B cell-mediated IgG immune responses, particularly for short peptide epitopes that exhibit intrinsically low immunogenicity (29, 30). Consequently, this study selected several self-assembling protein nanoparticles as carriers, including the AP205 phage capsid protein AP205 (31, 32), the 2,4-dioxotetrahydropteridine synthase Lus (33, 34), the hepatitis B virus core protein HBc (35, 36), and Ferritin (37, 38). These systems have been shown to form structurally ordered nanoparticles under *in vitro* conditions and possess strong immunostimulatory potential. To achieve efficient and site-directed antigen loading, we employed the SpyCatcher-SpyTag system. This system, derived from the internal isopeptide bond of the CnaB2 domain of the fibronectin-binding protein FbaB from Streptococcus pyogenes (39), has been utilized in the design of various vaccines (40, 41). Using this strategy, synthetic peptide antigens were covalently conjugated to the nanoparticle surface *in vitro*. Thus, SpyCatcher-fused nanoparticle carriers were expressed using a prokaryotic system, and two epitope peptides, SpyTag-Aβ29-35-3copy and SpyTag-Aβ36-42-3copy, were synthesized. Conjugation was conducted by incubating the carriers and peptides at a 1:4 molar ratio overnight at 4 °C (**Figure 4A**). Coomassie staining and Western blot analysis confirmed that all carrier proteins achieved a purity level exceeding 90% (**Figure 4B**). After overnight coupling at 4 °C, a minor upward shift in the electrophoretic bands of the conjugated nanoparticles was observed, indicating successful immunogen attachment to the nanoparticle carriers. The conjugation efficiency was estimated to be approximately 95%. Additionally, Western blot analysis corroborated the accurate presentation of peptides on the nanoparticle surfaces (**Figure 4C**). Furthermore, we also conducted a comprehensive characterization of eight conjugated nanoparticle types using transmission electron microscopy. The results revealed that the Ferritin-Aβ29-35-3copy and Ferritin-Aβ36-42-3copy nanoparticles exhibited superior spherical morphology, characterized by uniformly spherical particles. In contrast, the other six nanoparticles displayed a lower degree of sphericity (**Figure 4D**). Size distribution analysis further revealed that Ferritin-Aβ29-35-3copy and Ferritin-Aβ36-42-3copy nanoparticles consistently formed uniform particles with an average diameter of approximately 24 nm, demonstrating favorable stability. In contrast, nanoparticles utilizing AP205 or Lus as scaffolds exhibited sizes ranging from 15 to 25 nm, which were smaller than anticipated, suggesting suboptimal assembly efficiency. HBc-Aβ29-35-3copy nanoparticles showed a pronounced tendency toward aggregation, while the HBc-Aβ36-42-3copy nanoparticles failed to form intact spherical particles (**Figure 4E**). In summary, these findings demonstrate that Ferritin-based nanoparticles exhibit superior stability and assembly efficiency, making them a suitable platform for subsequent immunological evaluation and functional studies.

### 3.4 Nanoparticles exhibit favorable immunogenicity and safety profile

**Figure 5:**
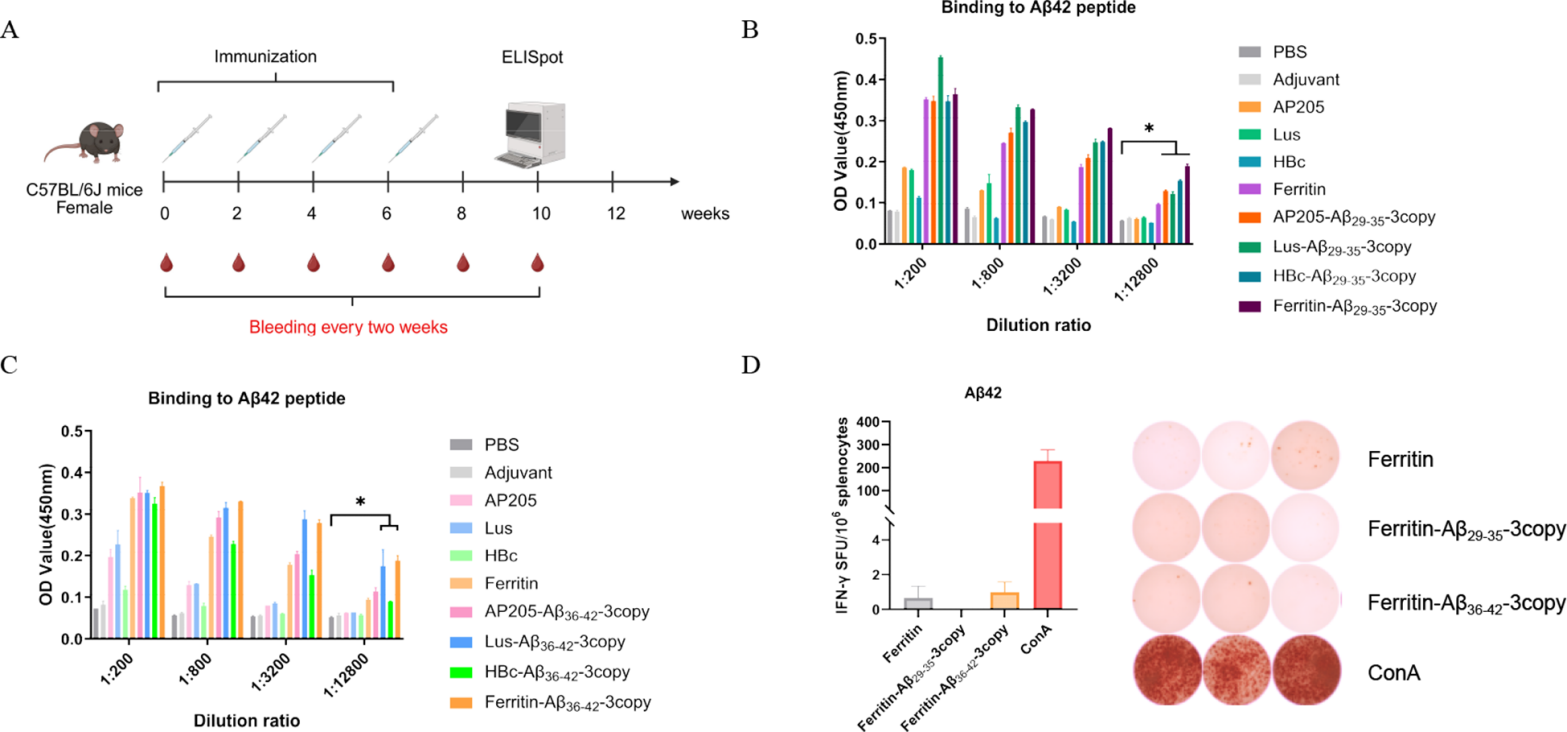
Evaluation of immunogenicity and safety of nanoparticle vaccines in C57BL/6J mice. **(A)** A schematic representation of the timeline for primary and booster immunizations, along with blood collection intervals, in WT C57BL/6J mice. **(B)** Quantification of Aβ-specific antibody titers after immunization with nanoparticles targeting Aβ29-35 using an ELISA. Asterisks (*) denote values that exceed twice the background absorbance, indicating the positive threshold. **(C)** Quantification of Aβ-specific antibody titers after immunization with nanoparticles targeting Aβ36-42 using an ELISA. **(D)** Analysis of T-cell activation. Aβ-specific T-cell responses were assessed using the IFN-γ ELISpot assay. Results are expressed as SFU per 10^6 splenocytes (n = 3).

The antibody immune response elicited by conjugated nanoparticles targeting specific C-terminal Aβ epitopes was evaluated in C57BL/6J wild-type mice following a four-dose immunization schedule. Serum samples were collected prior to each immunization, and vaccine-induced antibody levels were quantified via ELISA (**Figure 5A**). Serum antibody titers specific for the Aβ29-35 and Aβ36-42 epitopes demonstrated that all eight nanoparticle vaccines robustly induced epitope-specific antibody responses. Notably, the Ferritin-based vaccine generated the most pronounced anti-Aβ29-35 antibody response, with titers significantly exceeding those induced by other nanoparticle platforms (**Figure 5B**). Consistently, the Ferritin carrier also induced a stronger anti-Aβ36-42 antibody response compared to AP205 and HBc nanoparticles (**Figure 5C**). Due to the marked immunogenicity and high uniformity observed with the Ferritin-Aβ29-35-3copy and Ferritin-Aβ36-42-3copy nanoparticles, their safety was further investigated by assessing Aβ-specific T cell activation. According to ELISpot results, splenocytes isolated from mice immunized with these two Ferritin-based vaccines showed no detectable IFN-γ secretion upon Aβ antigen stimulation after the final immunization (**Figure 5D**), suggesting a lack of significant antigen-specific T cell activation. In conclusion, we successfully developed eight nanoparticle vaccines targeting the Aβ-ASC interaction site and systematically confirmed their efficacy in inducing target epitope-specific antibodies. Importantly, the Ferritin-based nanoparticles were distinguished by their ability to induce higher antibody titers, coupled with a favorable safety profile, improved particle homogeneity, and greater stability throughout the study, underscoring their promising potential for application.

### 3.5 Serum antibodies effectively recognize Aβ oligomers and pathological lesions in brain tissue sections

**Figure 6:**
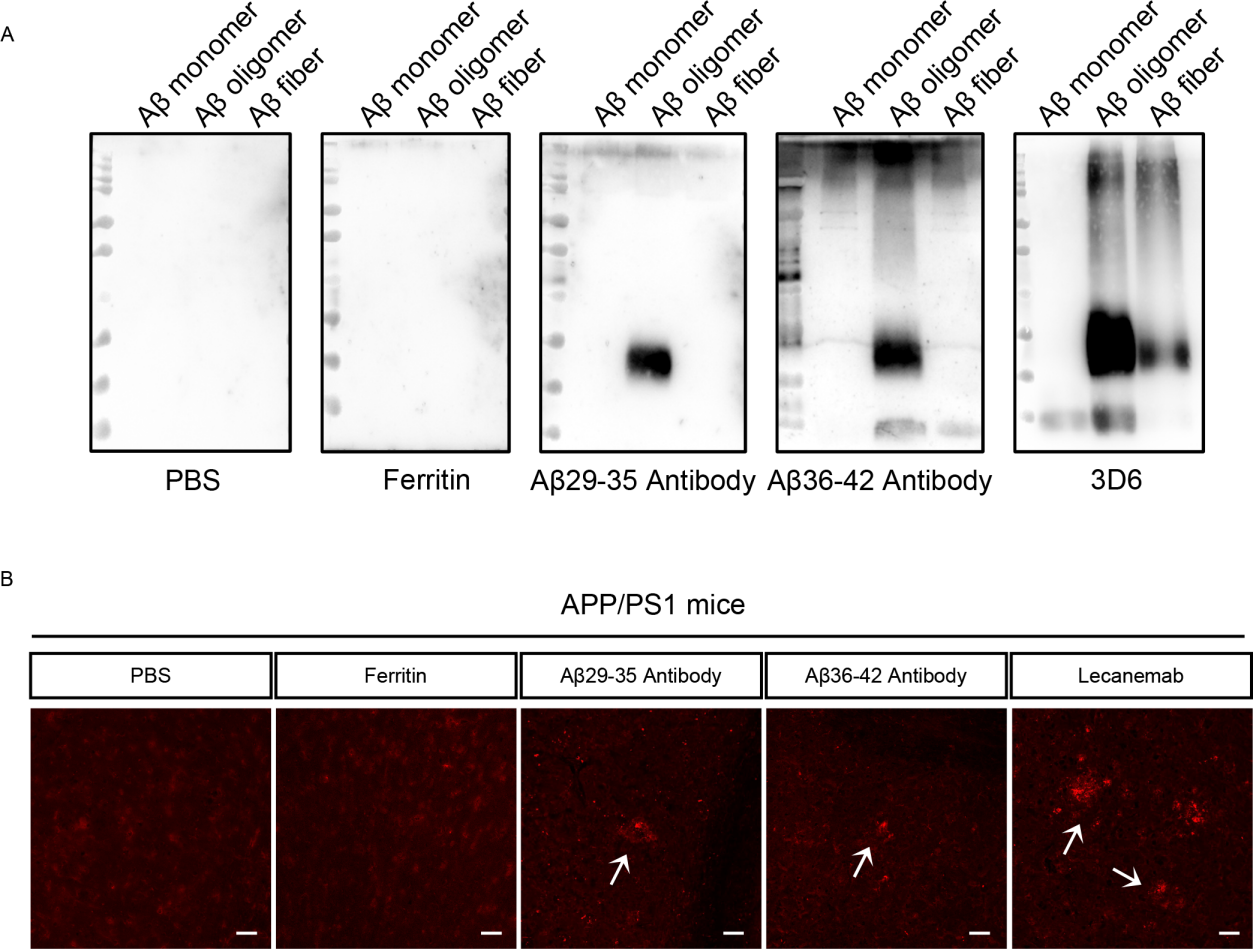
Recognition of Aβ isoforms and Aβ pathology by nanoparticle-generated antibodies. **(A)** Characterization of the recognition specificity of C-terminal antibodies against various forms of Aβ, as validated by Western blot. Five different antibodies were employed, including Aβ29-35 C-terminal antibody and Aβ36-42 C-terminal antibody. Negative controls consisted of PBS antibody and Ferritin carrier antibody, while the 3D6 antibody, which recognizes various forms of Aβ, served as the positive control. **(B)** Representative immunofluorescence images of Aβ pathology in the brains of APP/PS1 mice, detected with purified serum antibodies. The PBS and Ferritin nanoparticle immunization groups served as negative controls, while the Aβ-specific monoclonal antibody Lecanemab was used as a positive control (original magnification 20×, scale bars = 50 μm). White arrows indicate Aβ deposits.

Next, we systematically evaluated the specificity and targeting capacity of antibodies elicited by the Ferritin-Aβ29-35-3copy and Ferritin-Aβ36-42-3copy nanoparticle vaccines. To achieve this, specific antibodies were purified from the sera of immunized mice using saturated ammonium sulfate precipitation and subsequently tested for reactivity against various Aβ species. Purified serum antibodies from mice immunized with PBS or Ferritin alone showed no binding to any Aβ forms. In contrast, antibodies raised against Aβ29-35 and Aβ36-42 predominantly recognized neurotoxic Aβ oligomers (**Figure 6A**). To further assess the specificity of these antibodies toward Aβ-related pathology, we performed immunofluorescence staining on brain sections from 12-month-old APP/PS1 transgenic mice. The results indicated that antibodies induced by the two nanoparticle formulations, Ferritin-Aβ29-35-3copy and Ferritin-Aβ36-42-3copy, effectively labeled Aβ plaques in the brain, demonstrating high specificity for Aβ patholog. In comparison, serum antibodies from the PBS and Ferritin control groups showed no detectable binding to Aβ deposits in the brain tissues of AD model mice. Additionally, as a positive control, the Aβ-specific antibody Lecanemab clearly recognized Aβ pathology in the brain (**Figure 6B**). Collectively, these findings demonstrate that the nanoparticle vaccines targeting Aβ-ASC interaction sites can elicit specific serum antibodies that recognize neurotoxic Aβ oligomers and selectively bind to Aβ pathology in the brain, underscoring their strong targeting capability and pathological specificity.

### 3.6 Serum antibodies targeting the Aβ-ASC interaction interface effectively inhibit pathological binding and Aβ aggregation

**Figure 7:**
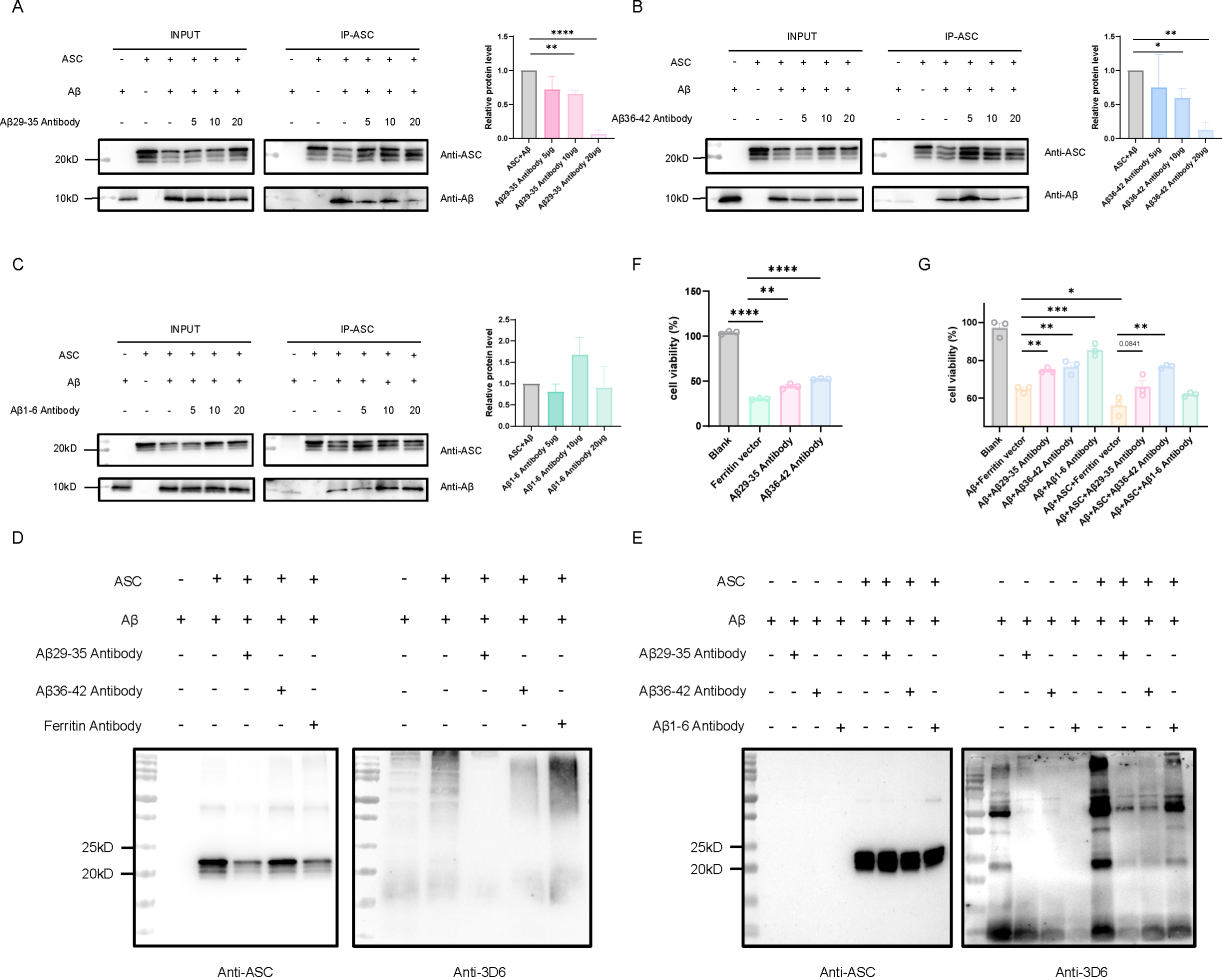
The impact of nanoparticle-generated antibodies on Aβ-ASC interaction and Aβ aggregation. **(A)** Evaluation of the antibody-mediated disruption of Aβ-ASC interaction. The inhibitory effect of the antibody targeting Aβ29-35 was detected by Western Blot (Left). Quantitative analysis of the inhibitory effect (Right). **(B)** The inhibitory effect of the Aβ36-42-specific antibody. **(C)** The inhibitory effect of the Aβ1-6 antibody. The data are presented as mean ± SEM (n = 3), and the data was analyzed using unpaired two-tailed Student’s t-tests, *p < 0.05, **p < 0.01, ****p < 0.0001. **(D)** The inhibitory effect of purified serum antibodies targeting Aβ C-terminal on Aβ aggregation, as validated by Western blot analysis. Three different antibodies were employed, including Aβ29-35 C-terminal antibody and Aβ36-42 C-terminal antibody, with a Ferritin carrier antibody used as the negative control. **(E)** The inhibitory effect of purified serum antibodies on ASC-induced Aβ aggregation was assessed using Western blot analysis. Three antibodies were used, including the Aβ29-35 and Aβ36-42 C-terminal antibodies, with the Aβ1-6 antibody serving as a control. **(F)** The protective effect of two Aβ C-terminal antibodies on Aβ oligomer-induced cytotoxicity. **(G)** The protective effects of two Aβ C-terminal antibodies and the Aβ1-6 antibody against ASC-enhanced Aβ aggregation-induced cytotoxicity, with the Aβ1-6 antibody serving as a control. The data are presented as mean ± SEM (n = 3), and the data was analyzed using unpaired two-tailed Student’s t-tests, *p < 0.05, **p < 0.01, ***p < 0.001, ****p < 0.0001.

To further investigate the therapeutic potential of blocking the Aβ-ASC interaction, we evaluated the ability of purified antibodies to inhibit their binding. In pull-down assays using full-length Aβ and ASC, various concentrations of purified antibodies were introduced to assess their effects on the interaction. The results demonstrated that antibodies targeting Aβ29-35 and Aβ36-42 significantly disrupted the Aβ-ASC association in a concentration-dependent manner, with higher antibody concentrations leading to stronger inhibition (**Figures 7A, B**). In contrast, control antibodies from mice immunized with Ferritin-Aβ1-6-3copy, which targets the Aβ1-6 epitope, showed no inhibitory effect, confirming the specificity of C-terminal Aβ antibodies in blocking Aβ-ASC binding (**Figure 7C**). To further investigate the inhibitory effects of these antibodies on Aβ aggregation, we performed *in vitro* aggregation assays. Consistent with previous reports, ASC protein significantly promoted Aβ aggregation. Notably, antibodies purified from the Ferritin-Aβ29-35-3copy and Ferritin-Aβ36-42-3copy immunization groups effectively inhibited Aβ aggregation, while antibodies from the Ferritin control group showed no such activity (**Figure 7D**). This indicates that these Aβ antibodies can suppress Aβ aggregation. To determine whether the observed inhibitory effects were due to the disruption of ASC specks-enhanced Aβ aggregation, we conducted another set of *in vitro* aggregation experiments. In the presence of Aβ alone, the Aβ1-6 antibody, known to inhibit Aβ aggregation, significantly reduced Aβ aggregation, similar to the Aβ29-35 and Aβ36-42 antibodies (42). However, in the presence of ASC plaques, the inhibitory effect of the Aβ1-6 antibody was markedly weaker than that of the Aβ29-35 and Aβ36-42 antibodies. These findings indicate that the Aβ1-6 antibody primarily inhibits Aβ self-aggregation, whereas the Aβ29-35 and Aβ36-42 antibodies suppress both Aβ self-aggregation and ASC specks-enhanced Aβ aggregation (**Figure 7E**). Next, we evaluated whether these specific antibodies could protect SH-SY5Y neurons from Aβ oligomer-induced cell death. Treatment with Aβ oligomers and a control antibody significantly reduced cell viability. In contrast, antibodies targeting Aβ29-35 or Aβ36-42 alleviated Aβ oligomer-induced toxicity and improved cell survival, with the Aβ36-42 antibody demonstrating a more pronounced protective effect (**Figure 7F**). To assess whether these antibodies also conferred neuroprotection in the presence of ASC, SH-SY5Y neurons were incubated with ASC specks and Aβ monomers for 24 hours. Compared with the Aβ+Ferritin vehicle group, the addition of ASC specks to the Aβ+Ferritin vehicle group resulted in greater cytotoxicity. Notably, our purified antibodies provided neuroprotective effects both in the presence of Aβ alone and when ASC specks and Aβ coexisted. Consistent with earlier findings, the Aβ1-6 antibody significantly reduced Aβ toxicity in the absence of ASC, but its protective effect was markedly diminished in the presence of ASC specks-Aβ complexes (**Figure 7G**). In summary, these findings indicate that Ferritin nanoparticle vaccines targeting key regions of the Aβ-ASC interaction can induce specific antibodies against C-terminal epitopes of Aβ. These antibodies not only effectively disrupt Aβ-ASC binding but also inhibit ASC-promoted Aβ aggregation. Furthermore, they reduce Aβ oligomer-induced neurotoxicity in a cellular model, supporting their potential therapeutic value for Alzheimer’s disease and laying a foundation for future therapeutic development.

## 4 Discussion

**Figure 8:**
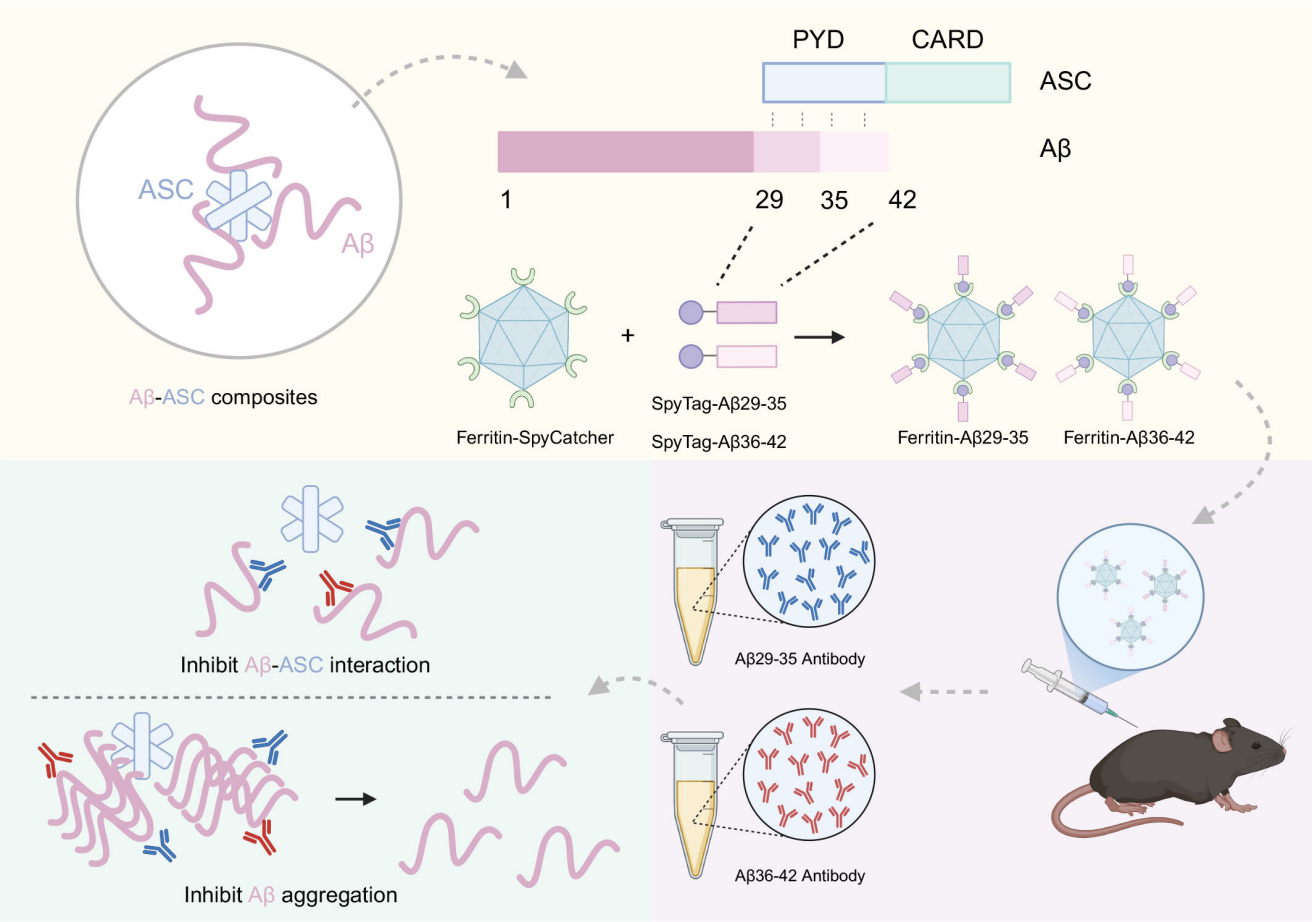
The effects of Aβ-ASC interaction on β-amyloid aggregation. The interaction site between ASC and Aβ is found within the amino acid residues 29-42 of Aβ. Nanoparticle vaccines targeting this site induce strong anti-Aβ antibody responses in mice. These antibodies not only block ASC’s binding to Aβ but also inhibit ASC-promoted Aβ aggregation. (Figure created with BioRender.com).

Over recent decades, the prevalence and mortality rates of AD have continued to rise alongside the aging global population. However, the pathogenesis of AD remains incompletely understood due to its complexity. Although several pharmacological treatments have been approved for AD, they can only partially alleviate symptoms and slow disease progression, rather than prevent or cure the disorder (43). Therefore, it is imperative to conduct an in-depth exploration of the underlying pathogenesis of AD and to identify effective therapeutic targets. In this study, we elucidated that the interaction between ASC and Aβ occurs within the 29-42 amino acid region of Aβ and the PYD domain of ASC. To further explore the functional significance of targeting this site, we engineered and purified specific nanoparticles conjugated with Aβ29-35 and Aβ36-42 immunogens, which exhibited strong immunogenicity in mouse models. Purified antibodies against Aβ29-35 and Aβ36-42 demonstrated a strong capacity for anti-aggregation and inhibition of cytotoxicity, underscoring the therapeutic potential of disrupting the Aβ-ASC interaction. These findings not only identify a novel target for immunotherapeutic strategies against Alzheimer’s disease but also support the development of inhibitors aimed at the Aβ-ASC interface. This approach may mitigate disease progression by reducing Aβ production and its associated neurotoxicity (**Figure 8**).

The NLRP3 inflammasome, a key sensor in the innate immune system, plays a central role in the neuroinflammatory pathology of Alzheimer’s disease (44). This multiprotein complex consists of the NLRP3 sensor protein, the apoptosis-associated speck-like protein adapter protein, and the pro-caspase-1 effector protein (45). Extensive studies have confirmed that the NLRP3 inflammasome contributes to AD pathogenesis by inducing chronic neuroinflammation and impairing the phagocytic functions of microglia and astrocytes (46). Specifically, in the brains of AD patients, NLRP3 inflammasome activation is closely associated with β-amyloid plaque deposition, hyperphosphorylation of Tau protein, and neuronal damage (47, 48). Microglia, the primary innate immune cells in the central nervous system, serve as the main site of NLRP3 inflammasome expression (49). In the presence of Aβ oligomers and fibrils, the NLRP3 inflammasome becomes activated in microglia, triggering neuroinflammatory responses (50). During this activation, the ASC protein interacts via its PYD domain with the PYD domain of NLRP3 and subsequently recruits pro-caspase-1 through its CARD domain, leading to the assembly of the inflammasome complex (51). This assembly ultimately results in the cleavage and activation of pro-caspase-1 into caspase-1, which promotes the maturation of pro-IL-1β and pro-IL-18 into IL-1β and IL-18, inducing pyroptosis (52). Furthermore, studies have shown that large perinuclear ASC specks form and are released extracellularly during NLRP3 inflammasome activation. These ASC specks can interact with Aβ and significantly promote its aggregation (53). However, the molecular basis of the Aβ-ASC interaction remains largely unexplored. In this study, we confirmed that ASC directly interacts with Aβ and markedly accelerates its aggregation using multiple experimental approaches. We identified the interaction site within the C-terminal amino acids 29-42 of Aβ. Deletion of either the Aβ29-35 or Aβ36-42 fragment severely disrupted the Aβ-ASC interaction. Further investigation revealed that the PYD domain of ASC is primarily responsible for the binding affinity to Aβ. Accumulating evidence indicates that the N-terminal region of Aβ is critical for its secondary structure formation and neurotoxicity (54), while the C-terminal region plays a central role in amyloid aggregation (55). Moreover, differences in oligomerization kinetics between Aβ40 and Aβ42 underscore the importance of C-terminal residues in Aβ aggregation (56). ASC functions as an adapter protein and contains both PYD and CARD domains (57). The PYD domain mediates the formation of ASC filaments, while the CARD domain facilitates the assembly of these filaments into dense ASC specks (58). Notably, point mutations in the ASC PYD domain completely abolished its ability to enhance Aβ aggregation. The distinct functional roles of the ASC and Aβ domains suggest that ASC filaments, formed through the PYD domain, may structurally resemble “seeds” for Aβ fibrils, thereby significantly accelerating Aβ aggregation. This aggregation process is primarily driven by the C-terminal region of Aβ. This mechanism offers a new perspective for understanding the molecular basis of Aβ aggregation in the pathophysiology of AD.

Over the past few decades, immunotherapy targeting Aβ has become a central strategy in the development of treatments for Alzheimer’s disease (59). This approach aims to clear abnormal Aβ aggregates from the brain to slow disease progression. One of the earliest attempts was the AN1792 vaccine, which used full-length Aβ42 as the antigen. In transgenic mouse models, the vaccine significantly reduced cerebral Aβ plaque load, demonstrating promising efficacy (60). However, the Phase II clinical trial was terminated due to limited effectiveness and serious adverse events. Specifically, only about 20% of patients developed a robust antibody response, and 6% of participants experienced meningoencephalitis (61). Subsequent studies indicated that these adverse effects were linked to T-cell epitopes in the C-terminal region of Aβ (62). To improve safety, later vaccine designs shifted to targeting B-cell epitopes in the N-terminal region of Aβ to avoid excessive T-cell activation (63). For example, ACC-001 used Aβ1-7 as the antigenic epitope, while CAD106 and AD02 targeted Aβ1-6. In addition, AD03 focused on the N-terminal pyroglutamate-modified Aβ, V950 selected Aβ1-15, and LU AF20513 was designed based on Aβ1-12 (64). Additionally, ABvac40 is a C-terminal-targeting vaccine that conjugates repeated Aβ33-40 fragments to keyhole limpet hemocyanin (KLH) to specifically recognize Aβ40, which is believed to play a significant role in AD pathogenesis, particularly in cerebral amyloid angiopathy (65). ABvac40 demonstrated a favorable safety profile in clinical trials, and Phase II results suggested it might slow disease progression and reduce global brain atrophy, although further data have not yet been released (66). Despite these advancements in safety, second-generation vaccines generally suffered from limited immunogenicity, leading to insufficient clinical efficacy and eventual discontinuation (67). This underscores the need for a deeper understanding of immune mechanisms to develop more effective AD vaccines.

In this study, to explore the impact of inhibiting Aβ-ASC interaction on Aβ pathology, we first developed nanoparticle-based vaccines targeting the C-terminal region of Aβ, using Aβ29-35 and Aβ36-42 as immunogens. To enhance the immunogenicity of the vaccine, we employed several types of nanoparticle carriers that have been demonstrated to be safe and effective in clinical trials. The results showed that we successfully purified four types of nanoparticle vectors and loaded the synthesized peptide corresponding to the ASC and Aβ binding epitope onto them with nearly 100% efficiency using the SpyCatcher-SpyTag system. Among the four carrier types, the Ferritin-based nanoparticles elicited the strongest immunogenic response against the Aβ C-terminal. Importantly, this response was not accompanied by the activation of Aβ-specific T-cell responses, indicating a favorable safety profile. These results imply that this target site exhibits significant immunogenic characteristics and an excellent safety profile, highlighting its potential as a therapeutic target for Alzheimer’s disease.

Notably, the purified serum antibodies showed high specificity for the highly toxic oligomeric forms of Aβ. These antibodies effectively suppressed the binding of ASC to Aβ in a dose-dependent manner. Most importantly, our findings demonstrated that the administration of specific antibodies significantly repressed Aβ aggregation, resulting in a marked reduction in its aggregated forms. Furthermore, our study showed that these antibodies could inhibit the neurotoxic effects of Aβ oligomers on neuronal cells. We propose that this neuroprotective effect is multifaceted. Previous studies have shown that antibodies targeting Aβ can reduce neurotoxicity by binding to soluble Aβ oligomers and protofibrils, preventing their interaction with specific receptors on the neuronal membrane (68). Additionally, we suggest that these purified antibodies recognize and bind to key interaction sites on Aβ, disrupting the Aβ-ASC interaction. This interference may hinder ASC’s role as a nucleation factor that promotes Aβ aggregation, ultimately leading to reduced Aβ accumulation and associated neurotoxicity. Moreover, in contrast to the Aβ1-6 antibody, which targets the N-terminus of Aβ, our Aβ C-terminal antibodies (Aβ29-35 and Aβ36-42) are directed against the interaction sites between ASC and Aβ. These antibodies not only inhibit Aβ self-aggregation and its associated neurotoxicity, similar to the Aβ1-6 antibody, but also further suppress ASC speck-enhanced Aβ aggregation and the resulting neurotoxicity.

In summary, our study clarified the interaction sites between ASC and Aβ, specifically identifying the amino acid region 29-42 of Aβ as the segment that binds to the PYD domain of ASC. Building on this finding, we used this epitope as an immunogen displayed on nanoparticle scaffolds to generate specific antibodies targeting the C-terminal region of Aβ in immunized mice. Specifically, the antibodies recognized neurotoxic Aβ oligomers and targeted pathological deposits in the brain tissues of APP/PS1 mice. Importantly, *in vitro* assays demonstrated that the purified antibodies effectively disrupted Aβ-ASC binding, and inhibited both Aβ aggregation and its associated cytotoxicity. Together, these results identify novel and effective targets and propose promising therapeutic strategies for the treatment of Alzheimer’s disease. Future studies should validate these findings in AD model mice and further investigate the therapeutic potential of blocking the Aβ-ASC interaction on neuropathology, cognitive function, and neuroinflammation.

## Data Availability

The original contributions presented in the study are included in the article/supplementary material. Further inquiries can be directed to the corresponding author.
